# Porous Carbon Fabricated by Microbial Pretreatment of Brewer’s Grain for the Improvement of Toluene Adsorption Performance

**DOI:** 10.3390/molecules29245931

**Published:** 2024-12-16

**Authors:** Jingxin Wang, Xiaohong Wang, Xiaoping Lin, Ziyi Yu, Davide Vione, Haomin Huang, Xiaohong Zhang, Yanhong Zhang, Jiaqi He, Yun Xia, Hansun Fang

**Affiliations:** 1 Guangdong Provincial Engineering Research Center of Public Health Detection and Assessment, School of Public Health, Guangdong Pharmaceutical University, Guangzhou 510310, China; 2 School of Art and Media, Guangzhou Vocational University of Science and Technology, Guangzhou 510550, China; wangxhcl@163.com; 3 Dipartimento di Chimica, Università di Torino, Via P. Giuria 5, 10125 Torino, Italy; 4 School of Environment and Energy, South China University of Technology, Guangzhou 510006, China; 5 Key Laboratory of Poyang Lake Basin Agricultural Resource and Ecology of Jiangxi Province, College of Land Resource and Environment, Jiangxi Agricultural University, Nanchang 330045, China

**Keywords:** microbial pretreatment, activated carbon, porous structure, toluene adsorption

## Abstract

Porous activated carbons (AC-AN and AC-AO) for toluene adsorption were prepared starting from brewer’s grain biomass pretreated with microorganisms (*Aspergillus niger* van Tieghem for AC-AN and *Aspergillus oryzae* RIB40 for AC-AO). The structures and chemical properties of the three activated carbon materials (AC-AN, AC-AO, and AC that was not pretreated with microorganisms) were characterized by N_2_ adsorption–desorption isotherms, scanning electron microscopy (SEM), transmission electron microscopy (TEM), Fourier-transform infrared spectroscopy (FTIR), Raman spectroscopy, X-ray diffraction (XRD), and X-ray photoelectron spectroscopy (XPS). The adsorption behavior of the three activated carbons for toluene was studied and correlated with the physical and chemical properties of these materials. The results suggested that the activated carbons prepared by microbial pretreatment had a rougher morphology, higher specific surface area, richer pore structure, fewer oxygen-containing functional groups on the surface, and better adsorption performance for toluene (increased by 31.5% and 18.3% with AC-AN and AC-AO, respectively) compared with the untreated activated carbon (AC). The Thomas model was used to fit the toluene adsorption data, indicating that the rich pore structure accelerated the kinetic process of toluene adsorption. Therefore, appropriate microbial pretreatment of the feedstock that is used to prepare activated carbon can effectively improve its adsorption capacity towards toluene.

## 1. Introduction

Volatile organic compounds (VOCs) are common pollutants in the petroleum and chemical industry, and these VOCs can cause global warming, destroy the ozone layer, and produce photochemical smog [1]. Some VOCs are teratogenic, carcinogenic, and/or mutagenic, and they pose serious harm to the environment and human health [2,3]. A lot of VOC control technologies are widely used [4]. Among them, adsorption is considered to be the most economical and effective technology to control a variety of VOCs, and the appropriate adsorbent is the key to adsorption technology.

However, the use of activated carbon (AC) to adsorb specific VOCs often does not allow for achieving the desired removal rate because of poor adsorption performance. The adsorption performance of VOCs on AC principally depends on the specific surface area, pore structure, and surface functional groups of the AC [5]. Some studies have reported that porous carbons with a high surface area generally show an excellent VOC adsorption capacity [6,7,8]. Furthermore, this high specific surface area mainly (~90%) comes from micropores that provide adsorption sites, while mesopores and macropores also play key role by accelerating the kinetic process of VOC adsorption and by promoting the migration and diffusion of VOCs [9,10,11]. Therefore, it is necessary to develop porous carbon manufacturing methods that produce ideal pore properties and are also economical and environmentally friendly, so as to improve the VOC adsorption performance.

A large number of studies have shown that various physical and chemical modifications of AC can increase the surface adsorption of VOCs and improve its removal ability [12,13,14,15]. However, the reported methods are expensive, require strict reaction conditions, and even cause secondary pollution to the environment. To find an economical and environmentally friendly modification method, the preparation of porous carbon materials with a high specific surface area by the microbial decomposition of biomass precursors has become a potential strategy. The microbial decomposition of lignocellulose (including cellulose, hemicellulose, and lignin) can degrade the internal structure of biomass and form a precursor with a loose structure that, combined with the carbonization and activation processes, allows for the construction of a large number of porous materials [16]. Therefore, microbial decomposition pretreatment may be an effective way to regulate the composition of biomass, produce a specific layered porous structure, and eventually provide excellent VOCs adsorbents. Some studies have reported the effect of the microbial degradation of biomass on the properties of carbon materials. For example, Deng [17] reported that the content of lignocellulose in biomass regulated the development of porosity and the surface characteristics of AC during heat treatment. Maple wood was cultured with *Phanerochaete chrysosporium* to carry out lignin degradation and form a large number of pores with a diameter of 2–5 nm [18]. In particular, it has been reported that the optimal ratio of pore size to VOC molecular size is between 1.7 and 3.0 for excellent adsorption performance [19]. Therefore, the applicability of various kinds of microbially pretreated hierarchical porous carbon precursors can be explored.

Most industrial and agricultural wastes (such as rice husk, bagasse, wheat straw, cotton stalk, and brewer’s grain) are directly discarded or burnt, which not only wastes resources but also causes harm to the environment. The waste materials mentioned above could become an important source of biomass precursors for lignocellulose materials, and they are also potential raw materials for the preparation of porous carbon for adsorption processes. In this study we used brewer’s grain (barley as raw material) as waste biomass precursor of a VOC adsorbent, in order to gain a high specific surface area, construct rich pores, and adjust the surface functional groups. The modified AC was prepared by decomposing and pretreating the brewer’s grain with *Aspergillus niger* van Tieghem and *Aspergillus oryzae* RIB40, respectively.

Common VOCs include formaldehyde, methanol, benzene and its derivatives, nitroaromatic hydrocarbons, aniline and benzo(a)pyrene, etc. Among them, benzene, represented by toluene, occupies a top position as an important raw material in house decoration, the chemical industry and surface coating [20]. Although the use of water-based solvents is gradually promoted in industry, the emission of toluene has also remained high. Toluene is considered to be the volatile organic compound that contributes most to ozone formation due to its high emissions and relatively high photochemical ozone generation potential [21]. Meanwhile, toluene can exist stably in the atmosphere in gaseous form due to its stable chemical properties, and it has irritating effects on the central nervous system, respiratory tract, and skin of human beings, which can easily cause poisoning or other diseases. Therefore, toluene was selected as the adsorbent in this study. For low and medium concentrations, atmospheric VOC (e.g., toluene) exhaust can be treated with microbially pretreated and non-microbially pretreated materials. This study provides support for the preparation of hierarchical porous carbon materials for VOC (e.g., toluene) removal, having a high specific surface area and rich pore structure, using a microbial pretreatment that is an economical and environmentally friendly modification method.

## 2. Results and Discussion

### 2.1. Characterizations of the Adsorbents

**Figure 1:**
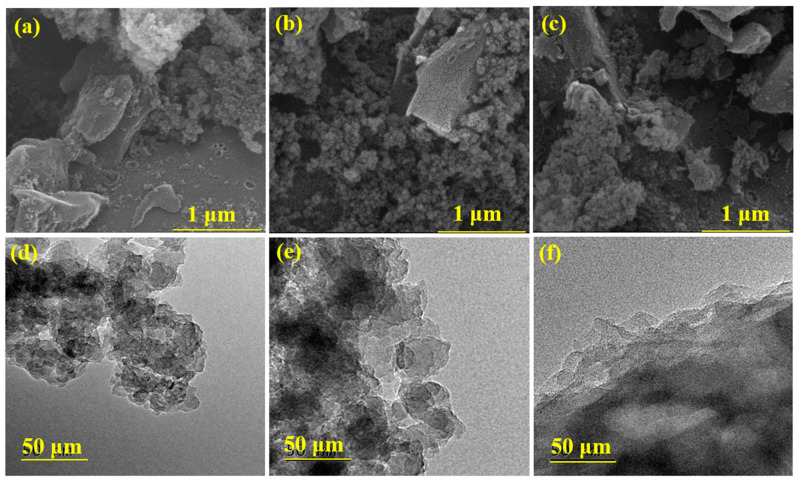
The morphology and structure of the ACs (AC (**a**,**d**), AC-AN (**b**,**e**), and AC-AO (**c**,**f**)) as studied by both SEM (**a**–**c**) and TEM (**d**–**f**).

The SEM images (Figure 1a–c) showed that the surface morphology of the ACs exhibited a rock-like shape, with an irregular and disordered size and shape of the porous structure.

However, compared to AC, the surfaces of AC-AN and AC-AO were rougher and looser, exhibiting more slit-shaped pores and disordered structures, presumably as a consequence of the microbial pretreatment of the original feedstock. These structures could be attributed to numerous micropores, mesopores, and macropores that formed hierarchical porous structures, potentially providing AC-AN and AC-AO with a high specific surface area. Hierarchical porous structures in activated carbons should also be favorable to VOC adsorption, as is often reported in the literature [22]. The growth and fine structure of the AC materials was further verified by TEM (Figure 1d–f and Figure S2); AC-AN and AC-AO samples contained plentiful pores and overlapping layers, contributing to the formation of hierarchical pores. The latter feature is linked with the presence of many micropores that are nested into the mesopores and macropores of the AC-AN and AC-AO materials.

Furthermore, AFM was used to characterize the three-dimensional porous structure of the Acs, because it can achieve dynamic three-dimensional imaging, thereby being an important tool for the analysis of carbon nanostructures. Three-dimensional AFM images showed that AC appeared as a block with a certain depth and a relatively smooth surface (Figure S1a), while the surfaces of AC-AN and AC-AO (Figure S1b,c) were filled with needle-like protrusions, generating a dense slit-type microporous/mesoporous structure. The results were similar to the AFM results of the microbiologically treated material [22]. In particular, there were more regular needle-like protrusions on the surface of AC-AN, which is conducive to the formation of micropores.

**Figure 2:**
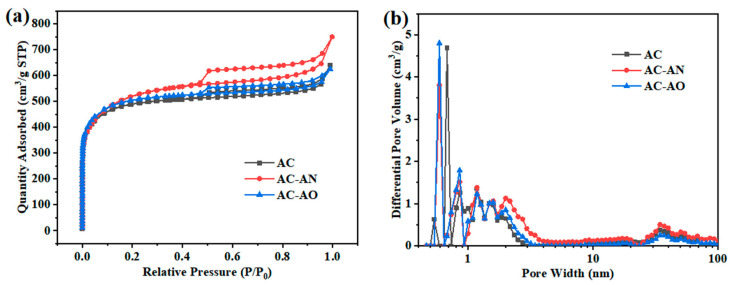
(**a**) The N2 adsorption–desorption isotherms and (**b**) pore-size distributions (PSDs) of ACs.

The above results suggested that biological feedstock pretreatment could enhance the formation of micropores, mesopores, and macropores in the relevant activated carbons, following lignocellulose decomposition by fungi. Figure 2 showed the N2 adsorption–desorption isotherms and the pore-size distributions (PSDs) of AC, AC-AN, and AC-AO. As shown in Figure 2a, all samples displayed combined type I and type IV isotherms with H4 hysteresis loops, based on the IUPAC classification [23]. The combined isotherms indicated that all the samples had a large number of microporous structures with partial mesopore contributions [24,25].

It should be noted that, when the relative pressure P/P0 was close to 1, the adsorbed quantity of N_2_ always increased to a certain degree, which suggested the presence of a few macropores in the materials. Therefore, all these samples showed microporous, mesoporous, and macroporous structures. After microbial degradation pretreatment, AC-AN and AC-AO had more pores of various grades, and in particular, AC-AN showed the largest hysteresis loops, which suggested an abundance of mesopores, and the results were similar to those of other studies [22]. The formation of mesoporous structures is compatible with the occurrence of a large number of internal migration channels, which is conducive to the migration of VOCs [26].

As shown in Figure 2b, all the samples showed a wide PSD that suggested the occurrence of micropores, mesopores, and macropores. In addition, compared with AC, AC-AN had more mesopores and macropores in the range of 2–100 nm, especially the most mesopores in the range of 2–4 nm, and AC-AO mainly had micropores of ~0.6 nm and ~0.86 nm, and it has been reported that the optimal ratio of pore size to pollutant molecule size needed to obtain excellent adsorption performance is as close to 1 (≥1) as possible [19] and that mesopores could accelerate the VOC adsorption kinetic process [9,10,11]. Therefore, AC-AN and AC-AO were more favorable for adsorption of more toluene molecules (0.67 nm). This might be due to the action of different microorganisms (*Aspergillus niger* van Tieghem and *Aspergillus oryzae* RIB40, respectively) on the raw material. *Aspergillus niger* van Tieghem expressed water-soluble proteins as well as organic acids better than *Aspergillus oryzae* RIB40; therefore, it is possible that the abundant secretion of enzymes as well as organic acids by *Aspergillus niger* led to the better treatment of beer seed biomass by *Aspergillus niger* van Tieghem than *Aspergillus oryzae* RIB40. Thus, it was shown that the microbially pretreated materials, especially AC-AN, had a better toluene adsorption potential.

Table 1 lists the specific surface area, microporous and mesoporous area, and total pore volume of the materials under study, showing that pretreatment by microorganisms (and especially *Aspergillus niger* van Tieghem, eventually yielding AC-AN) led to an increase in such quantities, presumably by decomposing the lignocellulose structure of brewer’s grain. A structural characterization suggested the successful preparation of ACs with a rich porous structure and highlighted the importance of selecting suitable microorganisms to obtain hierarchical porous carbon materials with a high specific surface area.

**Figure 3:**
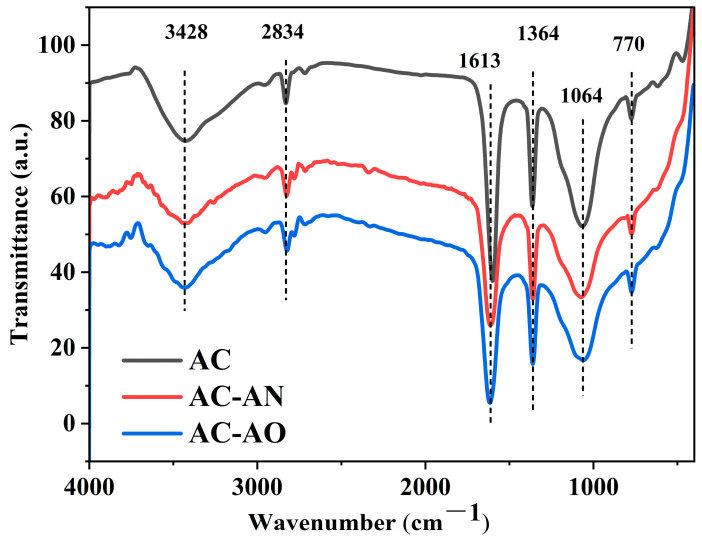
FTIR spectra of the investigated ACs.

Fourier-transform infrared (FTIR) spectroscopy was used to further study the changes in surface chemical properties of ACs with and without microbial treatment. It can be seen from Figure 3 that the type and position of the vibration peaks of the various samples were basically similar, but the intensity was quite different.

The broad band around 3700–3000 cm^−1^ had a peak at about 3428 cm^−1^ that corresponded to the stretching vibration of the hydroxyl functional groups (O-H). This was due to the inter- and intramolecular hydrogen bonding of alcohols, phenols, and carboxylic acids [27,28,29]. The small band around 3000–2800 cm^−1^ denoted the stretching of C–H in aliphatic hydrocarbons (-CH_3_, -CH_2_, -CH) [30,31]. Additionally, the band at 1613 cm^−1^ was assigned to the overlap between the v(C=C) stretching vibration mode of the aromatic ring carbon and the v(C=O) absorption bands of oxygen-containing groups [5,32,33]. The band at 1364 cm^−1^ was due to the C=O of carbonate groups, while the band at 1068 cm^−1^ belonged to the stretching vibrations of C-O in phenols or ethers [34,35,36]. The appearance of another small band at 770 cm^−1^ corresponded to an out-of-plane C-H in diversified aromatic structures, where the common band was between 900–700 cm^−1^ [37]. Compared with AC, a slight decrease in the absorption intensity of AC-AN and AC-AO could be observed for O-H, C=O, C-O, and other oxygen-containing functional groups. In contrast, the broad band of aliphatic hydrocarbons increased, while the out-of-plane C-H in diversified aromatic structures showed no significant change. The decrease in oxygen-containing functional groups is conducive to the adsorption of non-polar VOCs such as toluene [10,35], which might potentially be favored in AC-AN and AC-AO compared to AC.

**Figure 4:**
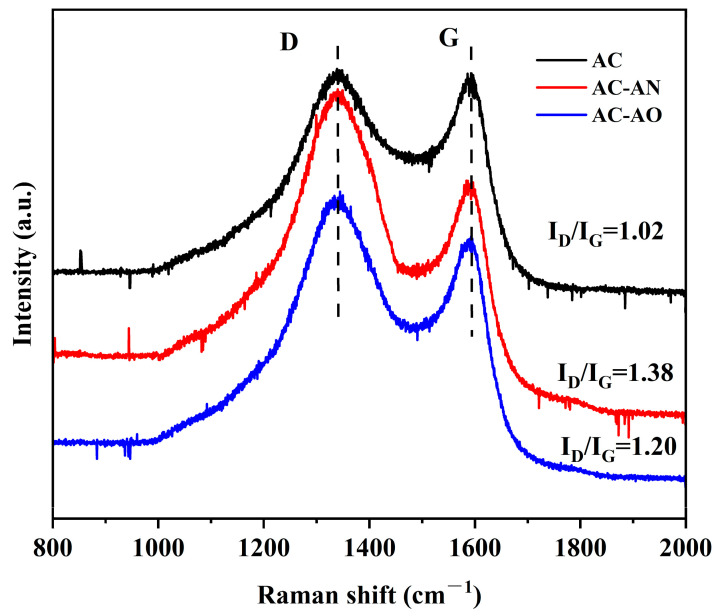
Raman spectra of ACs.

Figure 4 shows the Raman spectra of the studied ACs, which had two main characteristic peaks that included the D band (at 1340 cm^−1^) and the G band (at 1590 cm^−1^). Generally, the D band was related to disorder-induced defects of graphitic carbon, and the G band was associated with the in-plane vibration of sp^2^ C atoms [38,39]. The co-existence of two bands in the Raman spectra suggested partial AC graphitization with the introduction of defects and disorders [40]. The relevant intensity ratio of the D and G bands (ID/IG) could be applied to evaluate the degree of sample graphitization [22,26,41,42,43]: the higher the ID/IG, the higher the degree of irregularity. As shown in Figure 4 and Table S2, the calculated ID/IG values of AC, AC-AN, and AC-AO were 1.02, 1.38, and 1.20, respectively. Higher IG/ID values for AC-AN and AC-AO compared to AC suggested that the former had more defects and a more disordered structure, possibly linked with a higher porosity. Furthermore, the Raman spectra were fitted using a Gaussian model as shown in Figure S3. Shoulder peak I was related to the vibration of the aromatic rings. Peak II (D band) was related to the respiration vibration of the incomplete graphitic aromatic structures. Peak III was associated with the non-hexagonal rings. And peak IV (G band) was related to the in-plane vibration of sp^2^ hybridized carbon atoms. The appearance of peak V was due to the presence of carbonyl group in the sample. Therefore, the I_D_/I_G_ values calculated from the ratio of the areas of peaks II and IV could be used to estimate the degree of graphitization or defects in carbon samples [44,45,46]. The AC, AC-AN, and AC-AO values were 2.12, 2.95, and 2.36, respectively, which were in agreement with the results evaluated as the ratio of the relevant intensity ratio of the D and G bands.

The I_D_/I_G_ ratio was also inversely proportional to the crystal size of graphite [47], suggesting AC might have larger crystals that could be detrimental to VOC adsorption.

It is possible that, in the case of AC-AN and AC-AO compared to AC, the presence of more micropores, mesopores, and macropores (see Figure 2 and Table 1) could disrupt the graphite structure to some degree.

**Figure 5:**
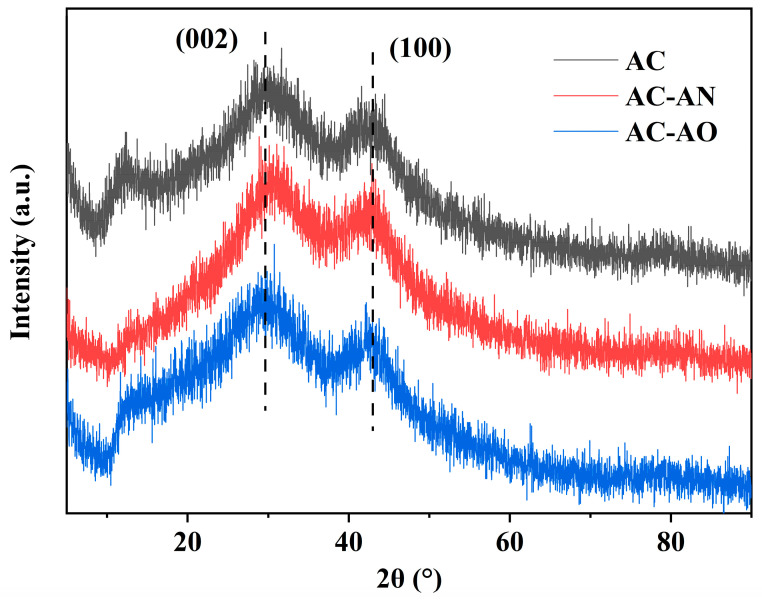
X-ray diffraction (XRD) spectra of ACs.

The XRD patterns of the AC samples are shown in Figure 5. Similar patterns were found in all of them, with two broad peaks at 28° and 43° that were related to the (002) and (100) planes, respectively, suggesting an amorphous carbon structure [48]. However, the peak intensity of AC-AN and AC-AO was higher than that of AC, indicating a higher extent of graphitization in the latter that was likely to be affected by the pore structure [43]. Additionally, the fact that the (002) plane of AC-AN and AC-AO was shifted to a lower angle compared to that of AC suggested a larger layer spacing in the case of AC-AN and AC-AO [42].

Generally, activated carbons derived from microbial feedstock pretreatment were more amorphous, thereby yielding more disordered samples with a higher S_BET_ that would be favorable to adsorption capability [49]. The XRD data were thus in good agreement with the results of the Raman.

**Figure 6:**
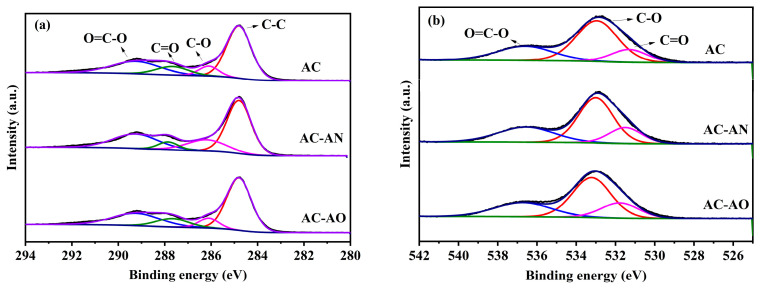
Curve-fitted C1s (**a**) and O1s (**b**) of XPS spectra for ACs.

XPS analysis was applied for evaluating the variation in the chemical bonding states, as well as the intensity of surface functional groups in the studied ACs, as shown in Figure 6 and Table S3. The XPS C1s survey spectrum of AC, AC-AN, and AC-AO had four obvious component peaks centered around 284.8, 286.2, 287.7, and 289.2 eV, which could be attributed to the C-C, C-O, C=O, and O=C-O groups, respectively [50,51]. According to the results of deconvolution, the proportion of C-O in AC-AN (9.3%) and AC-AO (8.4%) was lower compared to AC (10.6%).

Therefore, the XPS results suggested that the fewer C-O groups in AC-AN and AC-AO increased the surface electron density of these materials, which could enhance their π-π conjugated effect with VOCs [52]. In addition to this, it has been shown that the material contained fewer oxygen-containing groups, which facilitates the adsorption of nonpolar volatile organic compounds (e.g., toluene), and this result was in agreement with the FTIR results [10,35,53]. XPS spectra of O1s were also taken to verify the results of C1s XPS (Figure 6b). The XPS O1s spectrum could be separated into three peaks that consist of C-O (531.5 eV), C=O (533.4 eV), and O=C-O (536.9 eV) [54]. The shapes of the XPS data were similar among the three ACs, but the C-O groups were more numerous in AC (74.1%) compared to AC-AN (72.1%) and AC-AO (73.0%), which was consistent with the results of the C1s XPS.

### 2.2. Evaluation of Dynamic Adsorption Performance

**Figure 7:**
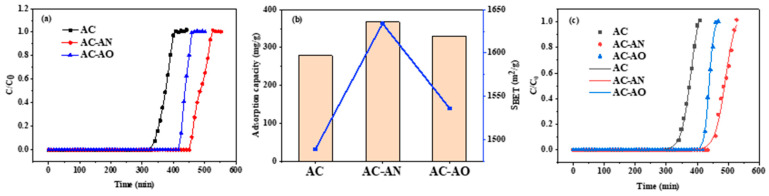
Breakthrough curves (**a**), the relationship of SBET and adsorption capacities (**b**), and the Thomas model (**c**) of ACs.

The ACs were evaluated for their adsorption performance towards gas-phase toluene using breakthrough measurement, which is a widely applied direct method to evaluate dynamic adsorption performance [10]. In the first phase, the breakthrough curves were approximately a straight line (see Figure 7a). Turning to the second phase, the outlet toluene concentration gradually increased after penetration.

When the sorption attained equilibrium, the inlet and outlet toluene concentrations were almost equal. The adsorption capacity of toluene on the ACs is reported in Table 2, which shows that the considerable right shift in the breakthrough curves of AC-AN and AC-AO with respect to AC (Figure 7a) affected the adsorption breakthrough time, which followed the order of AC-AN > AC-AO > AC. This finding means that the adsorption performance of AC-AN and AC-AO was better than that of AC. Moreover, among the investigated ACs, AC-AN showed the longest breakthrough time (454 min) and the largest adsorption capacity for toluene (367 mg/g) (Figure 7b). Compared to AC, the adsorption capacity of AC-AN and AC-AO increased by 31.5% and 18.3%, respectively (Table 2).

These different adsorption behaviors for toluene could be attributed to the different specific surface areas and surface functional groups of the investigated ACs, which largely affected the adsorption capacity [55]. Generally, adsorption capacity increased when increasing the specific surface area and the pore volume. The specific surface area typically had a peculiarly strong positive correlation with adsorption capacity, probably due to an increase in active site accessibility. Furthermore, structural properties, including pore volume, micro-porosity, and hierarchical pore structure, also affected the adsorption process [56]. In this framework, the increase in specific surface area and effect on surface functional groups that were linked with microbial feedstock pretreatment had a positive effect on toluene adsorption.

To understand the adsorption characteristics of toluene onto the investigated ACs, a semi-empirical model (the Thomas model) was used to fit the experimental data (see Figure 7c for the fitting curves), obtaining the adsorption kinetics parameters and the fitting accuracy that are listed in Table 2. The R^2^ values (>0.98) suggested very high correlations between the Thomas model and the experimental data. The constant *K_T_* measured the transfer rate of toluene from fluid to solid (i.e., from the gas phase to each relevant AC material), and the lower the value of *K_T_*, the higher is the mass transfer resistance [57,58]. The smallest *K_T_* value of AC-AN indicated that the sample had the largest mass transfer resistance, which was consistent with its relatively more microporous structure. The fitting trend can be seen more directly from Figure 7c. At the same time, AC-AN was the material with the highest value of maximum toluene adsorption capacity (Table 2), followed by AC-AO, which was consistent with the data of surface area (Table 1). Consequently, microbial feedstock pretreatment could significantly enhance the adsorption performance of the resulting activated carbon materials.

## 3. Materials and Methods

### 3.1. Preparation of Adsorbents

Air-dried brewer’s grain (Tsingtao Brewery (Sanshui) Co., Ltd., Foshan, China) was chosen as feedstock and exposed to the action of two different fungi, which were *Aspergillus niger* without spores (*Aspergillus niger* van Tieghem) and *Aspergillus oryzae* (*Aspergillus oryzae* RIB40), respectively, obtained from the Guangdong Microbial Strain Preservation Center (Guangzhou, China). The specific process was as follows: the cultured fungi fluid and spore fluid were inoculated in potato glucose (Aladdin Shanghai, Shanghai, China) fermentation medium (500 mL), to which air-dried brewer’s grain (15 g) was then added for pretreatment. The inoculation amount of *Aspergillus niger* van Tieghem was 3% (*v*:*v*, OD_600_ = 1) and that of *Aspergillus oryzae* RBI40 was 2% (*v*:*v*, spore concentration was 10^7^ cfu/mL). The potato glucose fermentation medium contained potato starch 10 g/L, glucose 20 g/L, KH_2_PO_4_ 5 g/L, and MgSO_4_·7H_2_O 0.5 g/L. Fermentation was carried out in a shaking incubator (DHZ-LA, Taicang Qiangle Experimental Equipment Co., Ltd., Taicang, China) at 30 °C and 200 rpm for 10 days. After fermentation and decomposition, the treated brewer’s grain was dehydrated by three layers of gauze and was then collected and washed with ultrapure water three times to remove the residual microorganisms. After cleaning, the brewer’s grain was dried at 80 °C until a constant weight for the later stage of preparation of activated carbon. In the latter procedure, the brewer’s grain was carbonized under N_2_ gas (99.99%) at 500 °C for 90 min with a heating rate of 5 °C/min. The product of carbonization was blended with KOH at a weight ratio of 2, and the mixture was heated under an N_2_ atmosphere at a rate of 5 °C/min from room temperature to 800 °C that was kept for 90 min. After cooling to room temperature, the brewer’s grain-activated carbon was mixed and stirred with 2 M hydrochloric acid solution (Guangzhou Chemical Reagent Factory, Guangzhou, China), rinsed with distilled water, and finally dried at 80 °C for 4 h. The activated carbon samples obtained by pretreatment with *Aspergillus niger* van Tieghem and *Aspergillus oryzae* RIB40 are here indicated as AC-AN and AC-AO, respectively. The activated carbon sample obtained without microbial pretreatment but with an otherwise identical procedure was named as AC. All samples were collectively referred to as ACs. Their activated carbon yields are shown in Table S1.

### 3.2. Characterization

The porosity of the ACs was characterized by N_2_ adsorption–desorption at 77 K (ASAP 2460, Micromeritics, Norcross, GA, USA). The Brunauer–Emmett–Teller (BET) method was used to calculate the specific surface area. Desorption branch isotherms obtained by the density functional theory (DFT) model were used to analyze the pore-size distribution (PSD). The surface morphological structures of the samples were characterized via scanning electron microscopy (SEM) using a JSM-7500F instrument (Jeol, Tokyo, Japan), by transmission electron microscopy (TEM) on a Tecnai F20 (FEI, Lausanne, Switzerland) instrument operated at 200 kV, and by atomic force microscopy (AFM, Dimension Edge, Bruker, Billerica, MA, USA). The surface functional groups were determined by Fourier-transform infrared spectroscopy (FTIR, American Thermo Fisher Scientific Company, Waltham, MA, USA). Raman spectroscopy was carried out with a microscopic confocal Raman spectrometer (LabRam HR800, Horiba, Kyoto, Japan). The crystalline properties were studied by X-ray diffraction analysis (XRD, D8 ADVANCE, Bruker). The chemical states were characterized via X-ray photoelectron spectroscopy (XPS, Thermo ESCALab 250, Waltham, MA, USA).

### 3.3. Adsorption Experiments

In order to evaluate the adsorption performance of AC, dynamic adsorption experiments were carried out at a temperature of 25 °C. Toluene, which is a typical VOC, was chosen as the target contaminant. The toluene vapor was produced through the bubbling method, and the toluene concentration in the vapor phase was measured with a mass flowmeter to be further adjusted. Nitrogen, used as a carrier gas, was applied to control the total flow rate (100 mL/min) to obtain the appropriate concentration (100 ppm for toluene). The gas concentration was measured by gas chromatography (GC-2014C, Shimadzu, Kyoto, Japan), and the adsorption capacity was calculated by the integral formula of the breakthrough curve according to the equation that follows:

(1) $$q=\frac{FC_{0}10^{-9}}{m}\left[t_{s}-\int_{0}^{t_{s}}\frac{C}{C_{0}}dt\right]$$

where *q* is the assessed maximum adsorption capacity (mg/g); *F* is the total gas flow rate (mL/min); *C*_0_ and *C* are the inlet and outlet concentrations of VOC (toluene), respectively (mg/m^3^); *m* is the weight of the adsorbing material (g); and *t_s_* is the adsorption time (min).

The Thomas model (Equation (2)) [57] was employed to simulate the breakthrough curves of the adsorbents:

(2) $$\frac{C}{C_{0}}=\frac{1}{1+exp(\frac{K_{T}qm}{Q}-K_{T}C_{0}t)}$$

where *C*_0_ and *C* (mg/mL) are the inlet and outlet VOC concentrations, respectively; *t* (min) is the adsorption time; *K_T_* (mL/(mg/min)) is the rate constant of the Thomas model; *q* (mg/g) is the estimated maximum adsorption capacity; *m* (g) is the mass of adsorbent; and *Q* (L/min) is the flow rate.

## 4. Conclusions

We prepared porous activated carbon derived from brewer’s grain biomass with a high toluene adsorption efficiency, by means of microbial decomposition pretreatment. The porous activated carbon materials thus prepared showed hierarchical porous structures, a high specific surface area, and large pore volume, as well as few oxygen-containing functional groups, which all could potentially improve the adsorption performance towards toluene. Correspondingly, the microbially pretreated materials showed superior adsorption performance compared to the activated carbon prepared without pretreatment.

This study has laid the foundations to explore the reliability of microbial pretreatment to prepare advanced porous carbon materials, with prospects towards practical applications. Actually, different microorganisms might be selected to enhance the targeted degradation of lignocellulose, or different raw materials for pretreatment. The goal would be the adjustment of the ratio of micropore to mesopore and the balance of mass transfer resistance, to obtain the most appropriate adsorption performance from these hierarchical porous carbon materials and, in the longer term, customized materials for toluene adsorption.

## Figures and Tables

**Table 1:** Pore structure parameters of ACs.

Name	S_BET_ (m^2^/g)	S_mic_ (m^2^/g)	Sext (m^2^/g)	S_mic_/S_BET_ (%)	Vtot (cm^3^/g)	V_mic_ (cm^3^/g)	Vext (cm^3^/g)	V_mic_/V_tot_ (%)	APD (nm)
AC	1489	1336	153	0.90	0.89	0.60	0.29	0.67	2.40
AC-AN	1634	1341	293	0.82	1.04	0.73	0.31	0.70	2.55
AC-AO	1536	1381	155	0.90	0.92	0.68	0.24	0.74	2.39

**Table 2:** Adsorption parameters and Thomas model fitting parameters of ACs.

Materials	Breakthrough Time (min)	Adsorption Capacity (mg/g)	Percentage Improvement in Adsorption Performance	Thomas Model		
				Estimated Maximum Adsorption Capacity (mg/g)	*K_T_* (mL/(mg/min))	R^2^
AC	335	279	-	311	158	0.998
AC-AN	454	367	31.5	403	143	0.989
AC-AO	419	330	18.3	360	316	0.997

## Data Availability

The original contributions presented in this study are included in the article/Supplementary Material. Further inquiries can be directed to the corresponding author(s).

## Supplementary Materials

The following supporting information can be downloaded at: https://www.mdpi.com/article/10.3390/molecules29245931/s1, Figure S1. AFM images of AC (a), AC-AN (b), AC-AO (c). Figure S2. TEM images of AC (a), AC-AN (b), AC-AO (c). Figure S3. Raman spectrum of AC (a), AC-AN (b), AC-AO (c). Table S1. Activated carbon yields of the studied samples. Table S2. Raman data of ACs. Table S3. XPS curve fitting results from high-resolution spectral scan for ACs.
