# Supplementary material for: Porous Carbon Fabricated by Microbial Pretreatment of Brewer’s Grain for the Improvement of Toluene Adsorption Performance
Source: Molecules. 2024 Dec 16;29(24):5931. doi: 10.3390/molecules29245931 (PMC11676505; doi:10.3390/molecules29245931)
Supplement: Supplementary file 1 [file molecules-29-05931-s001.zip › molecules-3339459-supplementary.pdf]

Porous Carbon Fabricated by Microbial Pretreatment of Brewer′s Grain for the Improvement of Toluene Adsorption Performance

Jingxin Wang [1,a], Xiaohong Wang [5,a] , Xiaoping Lin [1], Ziyi Yu [1], Davide Vione [3], Haomin Huang [4], Xiaohong Zhang [1], Yanhong Zhang [1], Jiaqi He [1], Yun Xia [1,*], and Hansun Fang [2,*]

[1] Guangdong Provincial Engineering Research Center of Public Health Detection and Assessment, School of Public Health, Guangdong Pharmaceutical University, Guangzhou 510310, China.
[2] Key Laboratory of Poyang Lake Basin Agricultural Resource and Ecology of Jiangxi Province, College of Land Resource and Environment, Jiangxi Agricultural University, Nanchang 330045, China.
[3] Dipartimento di Chimica, Università di Torino, Via P. Giuria 5, 10125 Torino, Italy.
[4] School of Environment and Energy, South China University of Technology, 510006, Guangzhou， China
[5] Guangzhou Vocational and Technical University of Science and Technology, 510555, Guangzhou， China
*Correspondence: fanghansun@163.com (H.F.); wangxhcl@163.com(X.W.); xy@gdpu.edu.cn (Y.X.).
[a]Jingxing Wang and Xiaohong Wang contributed equally to this work.

Supplementary Material

1. The process of obtaining brewer's grain (15 g) and the grain processing parameters of specific breweries and brewers.

(1) The beer grains were obtained from the fermented lees of the brewery, and the lees obtained from the brewery were first filtered to remove the water and washed with ultrapure water for 3 times, and then dried naturally. 15g of the dosage was based on the volume of Aspergillus co-cultivation to meet the shaking flask cultivation, and at the same time the dosage was to meet the preparation of biochar at a later stage.

(2) The brewery is Tsingtao Brewery (Sanshui) Co., Ltd, with an address of No. 38, Wenfeng West Road, Sanshui District, Foshan City, Guangdong Province. The raw materials for brewing and processing in the brewery are malt, hops, water, yeast and rice, of which the barley is imported from Australia. The grain processing parameters are (i) crushing: rice is crushed and then warm water at 45°C is added to make it swell and split; (ii) pasting and saccharification: crushed rice is added to the pasting oven, warm water is added and it is prepared into a complete liquid, then it is transferred to the saccharification oven and malt is added to saccharify it together; (iii) wort filtration: at the end of saccharification, the saccharified liquid is transferred to the filtration machine and the brewer's dregs and wort are obtained. The separated brewer's dregs are the initial brewer's dregs obtained in this article.

2. Preparation of samples for FTIR studies.

Sample preparation process: take the dried samples into the agate mortar with KBr particles and grind them evenly,

take a certain amount of samples for pressing, the pressed slices should not be too thin or too thick, and should have a certain degree of light transmittance.

3. Raman spectroscopy acquisition conditions.

The light source used was 532 nm with a scanning interval of 1000-2000 cm- 1. The spectrometer was equipped with a CCD (charge-coupled device) detector and a programmed temperature-controlled reaction cell (CCR1000). A He Ne laser ((λ = 532 nm)) was used, and the objective lens used to collect the Raman spectra was X50-05, the laser power was 50%, and the filter used was a RazorEdge filter LPD01. And the grating was chosen to be 1800 grooves mm- 1 for a single scanning time of 60 s, with a cumulative total of 2. The laser power was selected to be 6 mW.

4. Sources and wavelengths used for XRD data.

Cu Kα was used as the ray source (λ = 0.15418 nm), Ni filter, and the scanning area was from 5 to 60° with a minimum scanning step of 0.02°.

5. The XPS conditions of used excitation and calibrated spectrometer.

The binding energies of the elements were calibrated using Al Kα as the excitation source (hv= 1486.8 eV) and passing through the C 1s peak at 284.8 eV.

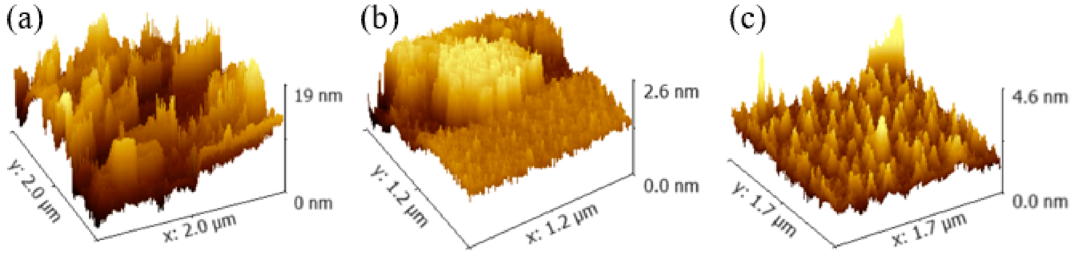

Figure S1. AFM images of AC (a), AC-AN (b), AC-AO (c).

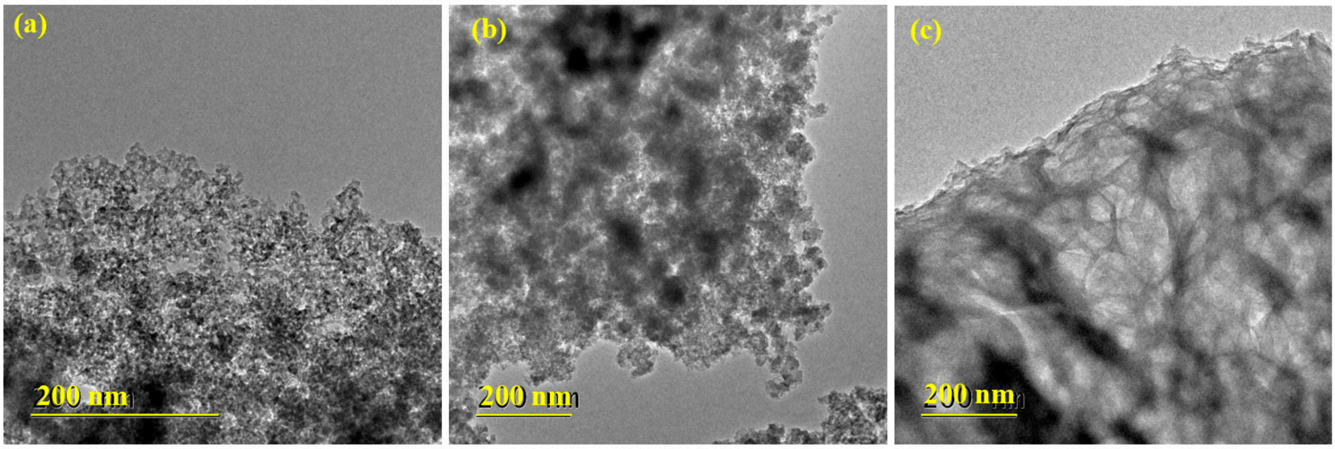

Figure S2. TEM images of AC (a), AC-AN (b), AC-AO (c).

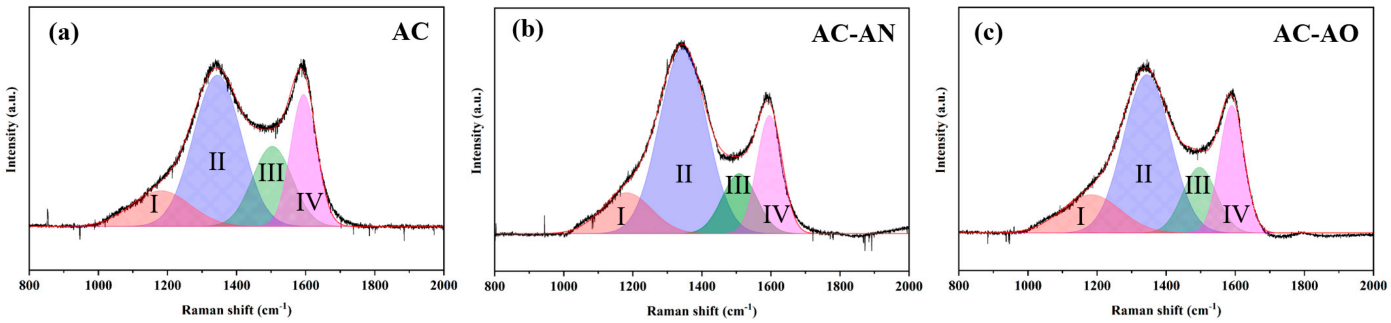

Figure S3. Raman spectrum of AC (a), AC-AN (b), AC-AO (c).

Table S1. Activated carbon yields of the studied samples

Sample	Activated carbon yield (%)
AC	16.78
AC-AN	17.84
AC-AO	16.45

Table S2. Raman data of ACs

Sample	I_D	I_G

AC	1641	1617
AC-AN	1556	1130
AC-AO	1126	939

Table S3. XPS curve fitting results from high-resolution spectral scan for ACs.

sample		name	position	fwhm	% at
AC	C1s	C-C	284.8	1.23	55.46
		C-O	286.2	1.21	10.61
		C=O	287.7	1.37	11.32
		O=C-O	289.2	1.71	22.61
	O1s	C=O	531.5	1.04	2.03
		C-O	533.3	2.92	74.08
		O=C-O	536.9	2.98	23.89
AC-AN	C1s	C-C	284.8	1.22	59.11
		C-O	286.1	0.98	9.31
		C=O	287.9	0.87	7.38
		O=C-O	289.2	1.77	24.20
	O1s	C=O	531.7	1.13	2.58
		C-O	533.4	2.59	72.13
		O=C-O	536.82	2.96	25.29
AC-AO	C1s	C-C	284.8	1.24	55.02
		C-O	286.1	1.00	8.36
		C=O	287.6	1.63	12.82
		O=C-O	289.2	1.87	23.81
	O1s	C=O	531.3	1.98	2.28
		C-O	533.4	2.89	73.03

	O=C-O	537.1	3.06	24.69
